# Fertility-Preserving Surgery of Borderline Serous Ovarian Tumors: A Case Report

**DOI:** 10.7759/cureus.24128

**Published:** 2022-04-13

**Authors:** Ipsita Mohapatra, Subha R Samantaray, Nikku Harshini

**Affiliations:** 1 Obstetrics and Gynecology, All India Institute of Medical Sciences, Kalyani, Kalyani, IND; 2 Obstetrics and Gynecology, Prathima Institute of Medical Sciences, Karimnagar, IND

**Keywords:** recurrence, younger women, conservative surgery, fertility sparing surgery, borderline ovarian tumors

## Abstract

Borderline ovarian tumors (BOTs) are tumors with low malignant potential and have an excellent prognosis. They are distinct by an epidemiological shift toward younger women. Fertility-sparing surgery is considered the gold standard in young patients presenting with BOTs. Spontaneous conception has been reported after conservative surgery with no enhanced risk of mortality or morbidity from disease progression during pregnancy.

The prognosis of BOTs is very good; however, a small proportion of these tumors may recur and show malignant transformation. Timely follow-up of the patients is required for timely detection of any recurrence.

We are presenting here a case of a 23-year-old woman diagnosed with BOT. The patient was nulliparous and hence was the appropriate candidate for fertility-sparing surgery. She underwent unilateral salpingo-oophorectomy and is now on regular follow-up.

## Introduction

Borderline ovarian tumors (BOTs) are tumors of low malignant potential. These represent tumor entity of epithelial origin, accounting for approximately 15%-20% of all primary ovarian neoplasms, and are characterized by atypical epithelial proliferation without stromal invasion [1,2].

They are marked by an epidemiological shift toward presenting women with young age and an excellent overall prognosis, with a five-year survival rate of more than 90% [3]. Since one-third of BOT patients are younger than 40 years, fertility-preserving aspects have become a substantial issue when treatment plan is being decided [3].

## Case presentation

A 23-year-old, married, nulliparous female presented with the chief complaint of intermittent dull aching pain in the right iliac fossa since three years. The patient had taken over-the-counter drugs for temporary relief of pain. But pain had become more intense since the past six days, and the patient was unable to carry out her daily chores. The pain was not associated with any alteration in bowel habits but was accompanied by occasional vomiting episodes. On general examination, the patient had normal vital signs. On gynecological examination, a well-defined mass of size around 7x8cm was palpable in the right iliac fossa. The mass was mobile with smooth margins, and there was no local rise in temperature. Mild tenderness was appreciated during palpation of the mass.

Routine blood investigations and ultrasonography were ordered. Transabdominal ultrasound revealed a unilocular cystic lesion measuring 88x69x74mm in the right adnexa and having solid component and papillary projections. These findings were confirmed on contrast-enhanced computed tomography (CT) of the abdomen and were suggestive of right ovarian serous cystadenocarcinoma. Apart from routine laboratory investigations, specific biomarkers were ordered. Carcinogenic embryonic antigen (CEA) was detected to be 0.771ng/mL and cancer antigen 125 (CA-125) was 195 U/mL.

**Figure 1:**
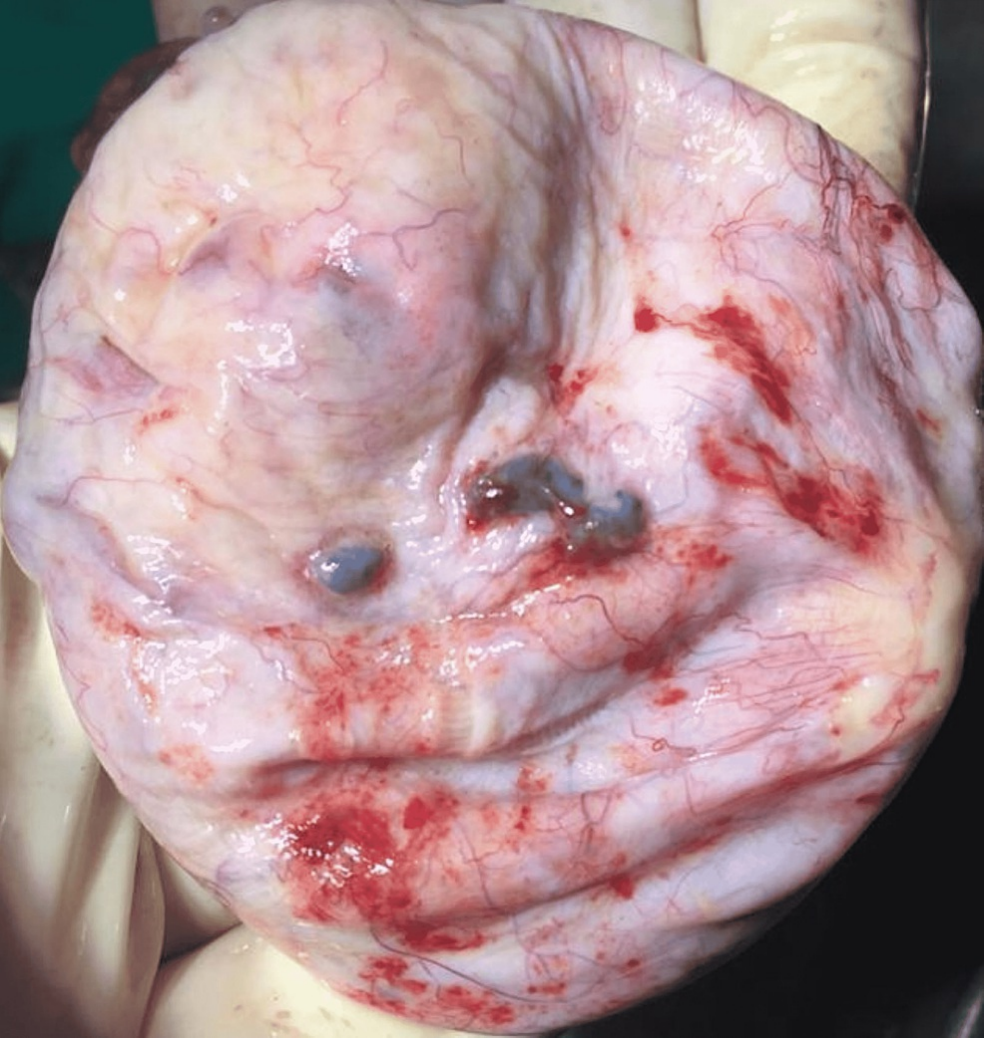
Smooth outer surface of the specimen

**Figure 2:**
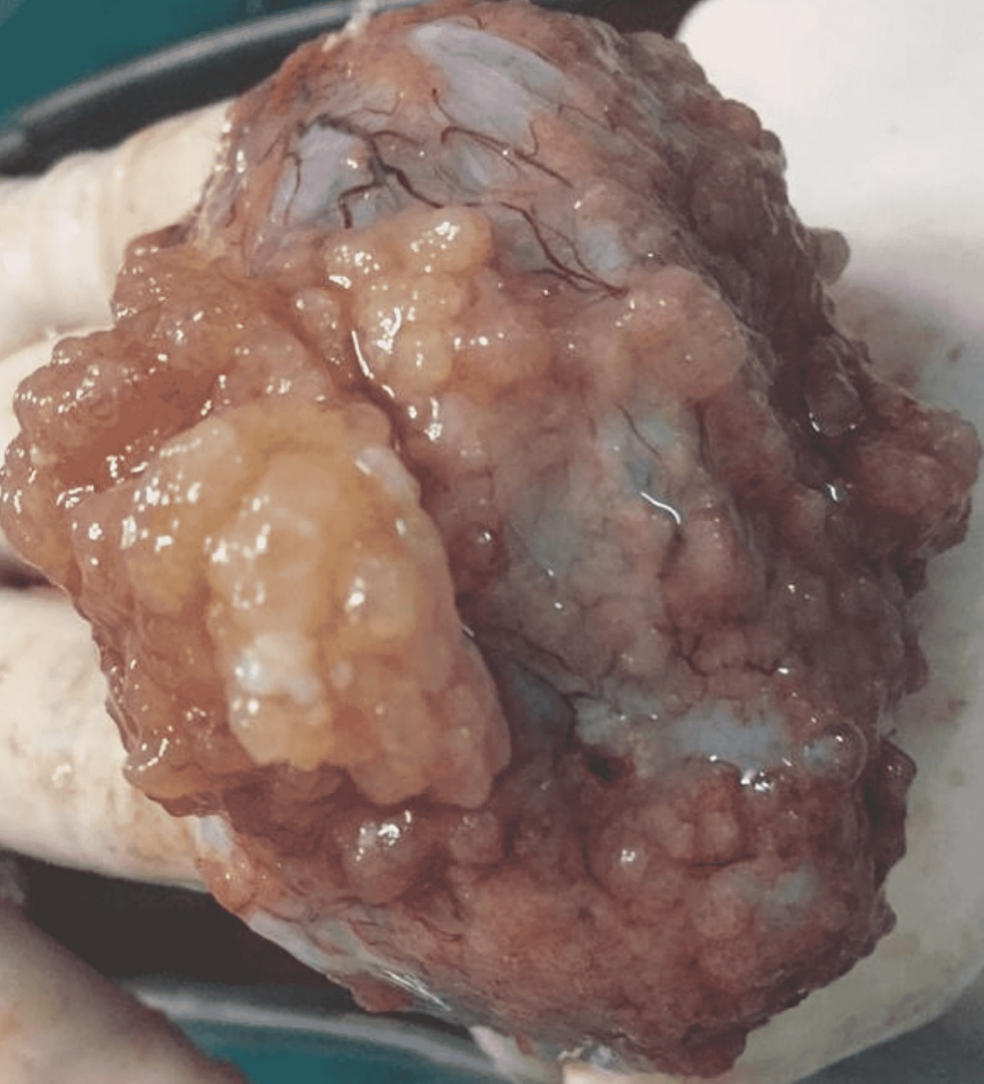
Cut section of the right ovarian tumor showing papillary excrescences on the inner surface

After completing the surgical profile and pre-anesthetic work-up, staging laparotomy was planned. The abdomen was opened by midline longitudinal incision. Peritoneal washings and systemic exploration of all intra-abdominal surfaces and viscera were performed. No signs of any implants or adhesions or metastasis were noted. Omental and peritoneal biopsies were taken. A smooth-surfaced 7x8cm unilocular cystic tumor arising from the right ovary was noted (Figure 1). Right-sided salpingo-oophorectomy was performed. The cut section of the specimen showed papillary excrescences arising from the solid part (Figure 2).

**Figure 3:**
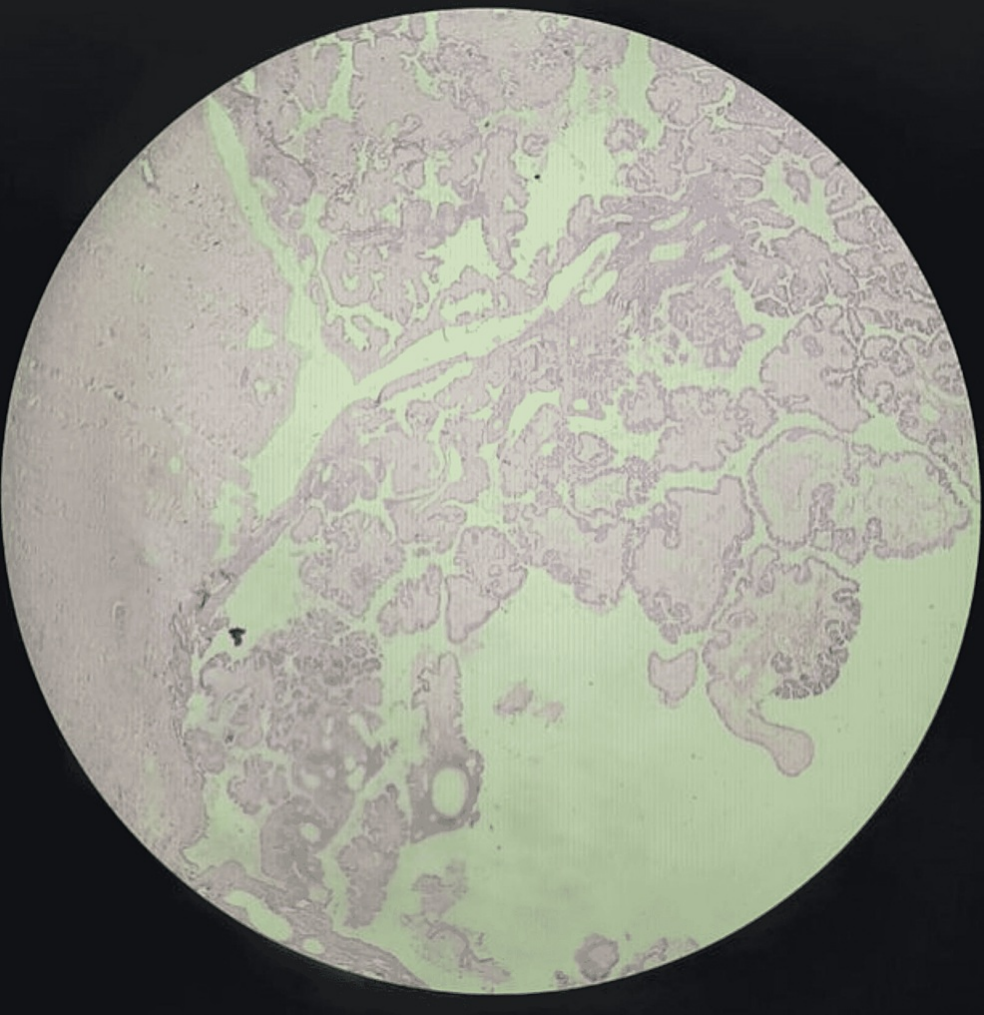
Histopathological slide (40x magnification) showing borderline ovarian pathology

The specimen was sent for histopathological examination. Definitive histopathological examination confirmed the diagnosis of borderline serous ovarian tumor without any evidence of stromal invasion (Figure 3). Post-operative period remained uneventful, and the patient was discharged on the seventh post-operative day.

Presently, the patient is being followed up at every six-month interval without any post-operative chemotherapy. At every follow-up, CA-125 and ultrasonography are repeated, and reports have shown no disease relapse.

## Discussion

BOTs are used to represent a group of epithelial ovarian tumors and were classified by the International Federation of Gynecology and Obstetrics (FIGO) in 1961 and adopted by the World Health Organization (WHO) in 1973 [4].

At present different nomenclatures are used for referring to this category of tumors, such as borderline tumor, tumor of low malignant potential, and atypical proliferative tumor [5]. BOTs comprise around 15%-20% of all epithelial ovarian malignancies [1,2], with an incidence of 1.8-4.8 per 100,000 women per year [6,7]. Out of these, serous borderline tumors (SBOTs) account for 50%-55%. This is followed by mucinous tumors (42.5%) and less common subtypes (4.2%) [8]. The less common subtypes of BOTs are endometrioid, clear cell, and transitional borderline tumors [9]. Around 50% cases occur in those younger than 40 years, and patients are often asymptomatic. The average age at presentation of the patients with borderline tumors is less than 40 years, and hence they are ideal candidates for fertility-sparing surgeries [2,4].

SBOTs are subclassified into atypical proliferative (90% with favorable prognosis) and non-invasive micropapillary type (5-10%) [10]. Ultrasonography remains the mainstay for evaluating the adnexa. BOTs differ from ovarian carcinomas due to the distribution of tumor subtypes, FIGO staging, better overall prognosis, presentation at a younger age, higher rate of infertility, and a lower association with *BRCA* mutations [4].

Most of the patients present in the early stages of the disease, and hence the prognosis is better. In a systemic review of 6,362 patients, 78.9% patients had FIGO stage 1 and 21.1% patients had stage 2-4, with stage 4 being very rarely present [8].

The prognosis of BOTs is very good. Around 11% of these tumors may recur, of which 20%-30% of cases may show malignant transformation [8]. Tumor size of more than 7cm, the presence of solid components and papillary structures, bilaterality, non-smooth margins, increased vascularity, and low resistance blood flow suggest malignancy (IOTA [International Ovarian Tumor Analysis] sensitivity > 90%). The CA-125 tumor marker does not appear to be useful in the early stages of these tumors. Most of these cases of BOTs have negative CA-125 [11]. The five-year survival rate for stage 1 BOT is around 95%-97%. The five-year survival for stage 2 and stage 3 patients is 65%-87% [12]. The features that can be related to poor prognosis are uncommon cell type, higher stage of the disease at presentation, implant type, and the presence of micropapillary architecture and any microinvasion.

In most ovarian carcinomas, fertility-preserving surgeries are performed only in some special cases, but in the case of BOTs, fertility-sparing surgery is considered the gold standard due to the young age at presentation [13]. Various treatment options such as unilateral cystectomy, unilateral oophorectomy, unilateral oophorectomy with contralateral cystectomy, and unilateral salpingo-oophorectomy can be opted for young patients desiring fertility preservation with minimal-to-moderate chances of recurrence. In a French multicenter study that included 313 patients with stage I BOTs, the recurrence rates were found to be 30.3%, 11%, and 1.7% after cystectomy, unilateral salpingo-oophorectomy, and bilateral salpingo-oophorectomy, respectively [14].

There is no recommendation for obtaining biopsy from the contralateral ovary as the risk of occult malignancy is extremely low [15]. In addition, taking biopsy may lead to formation of adhesions, which can have a negative impact on future fertility [16].

Spontaneous conception has been reported after conservative surgery in 50% of patients, with no enhanced risk of mortality from disease progression during pregnancy. Some studies have published pregnancy rates after treatment for early stage BOTs ranging from 30% to 80% [17,18]. Fertility treatments such as ovulation induction appear to be safe. But some studies have shown two- to threefold increased risk of BOTs after the use of ovulation-inducing drugs and ovarian drilling [19].

Chemotherapy is rarely indicated as a treatment option in BOTs. Regular follow-up of the patients is essential for the early detection of recurrence (usually in the spared ovary). The absolute rate for malignant transformation is around 2%-4% [4]. In a cohort study conducted by Uzan et al., it was observed that most of the non-invasive recurrence of BOTs could be diagnosed by ultrasonography. CA-125 elevation only occurred in case of progression to invasive ovarian cancers [20].

The important factors associated with disease recurrence are advanced age at the time of diagnosis, very high levels of CA-125 before treatment, and the presence of invasion and micropapillary pattern on histology [21]. BOTs have excellent overall survival rates, as shown in Table 1 [7].

**Table 1:** Borderline ovarian tumors survival rate [7] FIGO, International Federation of Gynecology and Obstetrics

Stage (FIGO)	5-year rate (%)	10-year rate (%)
I	99	97
II	98	90
III	96	88
IV	77	69

## Conclusions

BOTs represent a wide spectrum of tumors with different biological potential and uncertain malignant potential. They occur mainly unilaterally, are diagnosed at an early stage, and present a good overall prognosis. They are difficult masses to correctly classify preoperatively as their macroscopic features overlap with invasive and benign ovarian tumors. Though this category of tumors is not uncommon, the issue of fertility preservation is important. Conservative surgery can be proposed to young patients with a future child-bearing under careful follow-up.
